# Cancer-mutated ribosome protein L22 (RPL22/eL22) suppresses cancer cell survival by blocking p53-MDM2 circuit

**DOI:** 10.18632/oncotarget.21544

**Published:** 2017-10-06

**Authors:** Bo Cao, Ziling Fang, Peng Liao, Xiang Zhou, Jianping Xiong, Shelya Zeng, Hua Lu

**Affiliations:** ^1^ Department of Biochemistry & Molecular Biology, Tulane Cancer Center, Tulane University School of Medicine, New Orleans, LA, USA; ^2^ The First Affiliated Hospital of Nanchang University, Nanchang, People’s Republic of China; ^3^ Fudan University Shanghai Cancer Center and the Institutes of Biomedical Sciences, Fudan University, Shanghai, People’s Republic of China

**Keywords:** RPL22/eL22, p53, MDM2, RPL11/uL5, RPL5/uL18

## Abstract

Several ribosomal proteins (RPs) in response to various ribosomal stressors have been shown to play a critical role in p53-dependent regulation of cell cycle arrest, apoptosis and tumor suppression. Here, we report ribosomal protein L22 (RPL22/eL22) as a novel p53 activator highly mutated (mostly deletion mutation) in various types of human cancers, but not essential for ribosomal biogenesis in normal cells. Ectopic expression of RPL22/eL22 suppressed the colony formation of cancer cells in a p53-dependent manner, whereas knockdown of RPL22/eL22 significantly compromised p53 activation by Actinomycin D, rescuing p53-induced G1/G0 cell cycle arrest. Interestingly, human tumors with RPL22/eL22 deletion appeared to sustain wild type p53. Mechanistically, RPL22/eL22 bound to MDM2 acidic domain and inhibited MDM2-mediated p53 ubiquitination and degradation, hence extending the half-life of p53. Ribosome-profiling analysis revealed that induction of ribosomal stress by Actinomycin D leads to the increase of ribosome-free RPL22/eL22 pool. Also, RPL22/eL22 formed a complex with MDM2/RPL5/uL18/RPL11/uL5 and synergized with RPL11/uL5 to activate p53. Furthermore, the N terminus of RPL22/eL22 bound to MDM2, while the C terminus interacted with RPL5/uL18/RPL11/uL5; both of these two fragments activated p53 by inhibiting MDM2. Our study indicates that RPL22/eL22 highly mutated in human cancers plays an anti-cancer role likely through regulation of the MDM2-p53 feedback loop, and also suggests that targeting the RPL22/eL22-MDM2-p53 pathway could be a potential strategy for future development of anti-cancer therapy.

## INTRODUCTION

Tumor cells utilize gene mutations to alter cell signal transduction events in favor of cell survival. Genomic sequencing analysis of multiple types of human cancers revealed that the gene encoding the large subunit ribosome protein L22 (RPL22/eL22, all ribosome proteins will be named according to both old and new systems described in a review paper [1]) is highly mutated in endometrial carcinoma [2]. The high mutation rate of RPL22/eL22 was also confirmed in other studies on T-acute lymphoblastic leukemia (T-ALL), endometrial and colorectal carcinomas[3, 4]. A latest report showed that RPL22/eL22 is the most recurrently mutated/deleted ribosomal protein gene (RPG) in 30 cell lines with intact and functional p53, differing from other RPGs that are more likely deleted in *TP53*-mutated tumors in a large-scale analysis of human cancer genome data [5]. These studies suggest that RPL22/eL22 is a potential tumor suppressor. It has been reported that inactivation of RPL22/eL22 facilitates T-lineage progenitors to transform by inducing the expression of stemness factor LIN28B [4]. Although it has also been shown that RPL22/eL22 deficiency leads to the activation of the tumor suppressor p53 in immune cells, and RPL22/eL22 may mediate Trp53 translation via Miz1 in cells undergoing V(D)J recombination [6, 7], it still remains unknown if RPL22/eL22 might play a possible tumor suppression role by regulating the p53-MDM2 loop in response to ribosomal stress in non immune cells since RPL22/eL22 is ubiquitously expressed in all cells and tissues.

TP53 that encodes p53 is one of the most important tumor suppressor genes and mutated in approximately 50% of all human cancer types [8, 9]. The rest of the cancers harbor wild type p53 that is often inactivated due to enhanced p53 antagonistic functions or silenced p53-activating pathways [8-10]. An important p53-controlling molecule often highly expressed in some cancers is MDM2 that inhibits p53 activity by directly binding to it and mediating its ubiquitin-dependent proteolysis, as MDM2 possesses intrinsic E3 ubiquitin ligase activity [11, 12]. Previous studies including ours and others’ have shown that ribosomal stress triggered by disturbing ribosome biogenesis can lead to p53 activation, mainly by enhancing the binding of ribosome-free ribosome proteins (RPs) with MDM2, consequently inhibiting MDM2 E3 ligase activity toward p53 [13-16]. These p53-activating ribosomal proteins include RPL11/uL5, RPL5/uL18, RPL23/uL14, RPL26/uL24, RPS7/eS7, and RPS14/uS11 [13, 14, 17-22]. Thus, we were initially inspired to find out if RPL22/eL22 might also be involved in ribosomal stress induction of p53, as the RPL22/eL22 mutation rate (mostly deletion) is considerably high in primary cancers [2, 3].

Indeed, we found that RPL22/eL22 is required for ribosomal stress induction of p53, and the mutation statuses of RPL22/eL22 and p53 are mutually exclusive to each other in human cancers. Interestingly and mechanistically, the N- and C-termini of RPL22/eL22 played distinct roles in inhibiting MDM2. Our study as presented here not only unveils RPL22/eL22 as a novel p53 activator, but also provides new insight into the mechanisms underlying the activation of p53 by this cancer-mutated RP as well as a reasonable and molecular interpretation for why RPL22/eL22 is highly mutated in several human cancers.

## RESULTS

### RPL22/eL22 is highly mutated in human cancers

**Figure 1:**
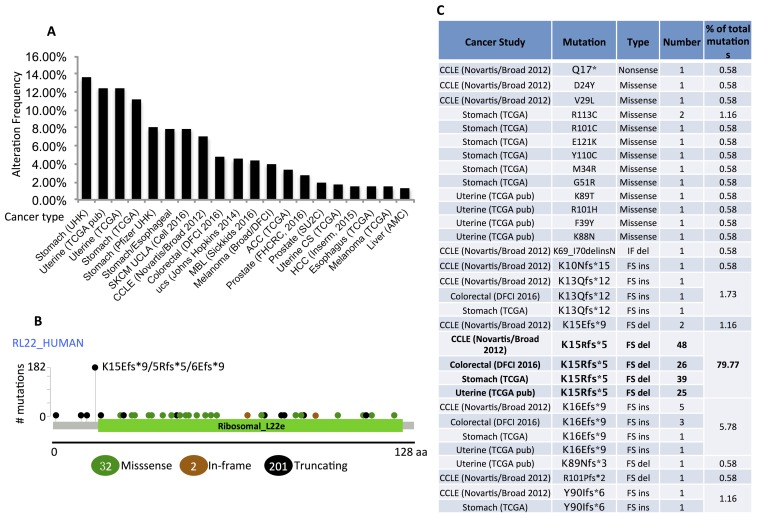
RPL22/eL22 is highly mutated in human cancers **A.** Mutation rates of RPL22/eL22 in human cancers. **B.** Mutation sites of RPL22/eL22 in the top four RPL22/eL22 mutated human cancers. **C.** Details of RPL22/eL22 mutation types in the top four RPL22/eL22 mutated human cancers. The most frequent mutation type K15Rfs*5 was highlighted in Bold. FS: frame shift; del: deletion; ins: insertion. All data were retrieved from cBioPortal.org.

Previous studies showed that RPL22/eL22 is highly mutated in endometrial and colorectal carcinomas [2, 3]. By exploring human cancer databases available in cBioPortal (Supplementary Figure 1) [23, 24], we found that in addition to in endometrial and colorectal tumors (∼5%-12%), RPL22/eL22 is also highly mutated in stomach cancer (∼8%-13%) and a pool of cancer cell lines (Cancer Cell Line Encyclopedia, CCLE, Novartis/Broad, ∼7%) (Figure 1A). Included in Figure 1A are other cancer types in which the mutation rate of RPL22/eL22 ranges from ∼1% to 4%. Interestingly, when we looked into the mutation types, we found that 201 out of 235 mutations were truncating mutations, among which 186 frame shift deletions occurring at the N terminus of RPL22/eL22 (Figure 1B). The examination of detailed RPL22/eL22 mutations in the top four RPL22/eL22 mutated cancers revealed that the most frequent mutation—K15R frame shift accounts for 79.77% of all the mutations (Figure 1C). Since RPL22/eL22 is not an essential ribosome protein for protein translation [25] and cell growth [6], these data suggest a potential tumor suppression role of RPL22/eL22 in human cancers.

### RPL22/eL22 inhibits cancer cell colony formation by activating p53

**Figure 2:**
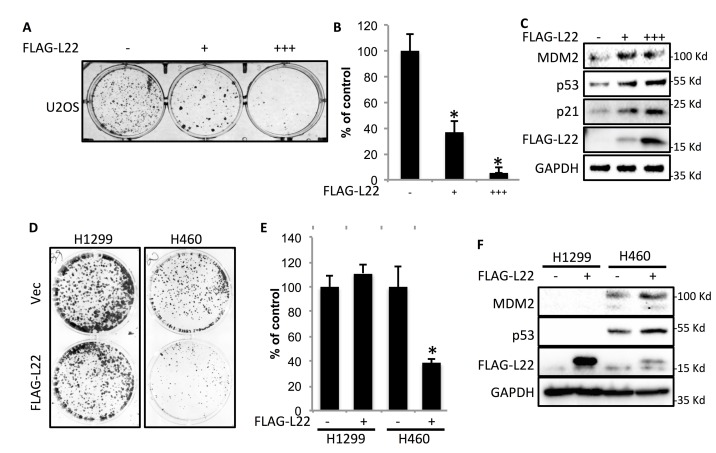
L22 suppresses cancer cell colony formation in a p53-dependent manner U2OS cells were transfected with pcDNA3.1 or increasing amount of FLAG-L22. 24 h later the transfectants were replated in 6-well plates at 2000 cells/well. **A**-**B**. G418 selection was performed for 10 days and the colonies were visualized using crystal violet staining and colony numbers were counted and plotted. **C**. FLAG-L22 expression and p53 pathway activation were confirmed by WB analysis. H1299 and H460 cells were infected with lentivirus pLenti6-Vec or pLenti6-FLAG-L22 and subjected to blasticidin selection for 10 days. **D**. The colonies were visualized using crystal violet staining. **E**. Colony numbers were counted and plotted. **F**. FLAG-L22 expression and p53 pathway activation were confirmed by WB analysis. *, *p*<0.05 as compared to vector control. All experiments were performed in triplicate.

To determine if RPL22/eL22 could negatively affect cancer cell growth, we transfected osteosarcoma cell line U2OS with increasing amounts of the FLAG-L22 plasmid with an empty vector as a control, and counted the number of colonies after 10 days of selection with G418 treatment. The results clearly showed a dose-dependent inhibition of colony formation by ectopic FLAG-L22 (Figure 2A & 2B). Interestingly, when examining the expression of FLAG-L22 and p53 as well as some of p53’s target genes by Western blot (WB) analysis, we found that overexpression of RPL22/eL22 leads to the increase of p53, p21 and MDM2 protein levels. These results suggest that RPL22/eL22 might inhibit cancer cell growth by activating p53. Next, we checked if RPL22/eL22 can lead to the p53-dependent suppression of cancer cell colony formation by using two sets of cancer cell lines, including lung cancer (H1299^p53-/-^ and H460^p53+/+^) and colon cancer (HCT116^p53-/-^ and HCT116^p53+/+^) cell lines. As shown in Figure 2D and 2E as well as Supplementary Figures 1A and 1B, overexpression of RPL22/eL22 significantly suppressed colony formation of p53 positive, but not p53 negative, cells, although the expression level of ectopic RPL22/eL22 was relatively lower in p53 positive cells than that in p53 negative cells (Figure 2F and Supplementary Figure 1C). Collectively, these results demonstrate that RPL22/eL22 can suppress cancer cell proliferation and growth in a p53-dependent fashion.

### RPL22/eL22 is required for ribosomal stress induction of p53

**Figure 3:**
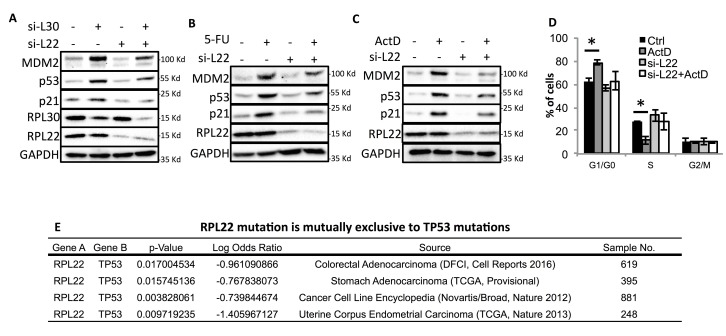
L22 is required for ribosomal stress-induced p53 activation **A.** U2OS cells were transfected with scramble siRNA control (-), or si-L30 with or without si-L22 and subjected to WB analysis 72 h after transfection. **B** & **C**. U2OS cells were transfected with scramble control siRNA or si-L22. 48 h after transfection, the cells were treated with 10 μg/ml 5-FU (B) or 5 nM Actinomycin D (ActD) (C) for 18 h followed by WB analysis with antibodies as indicated. **D**. U2OS cells were transfected with scramble control siRNA or si-L22. 48 h after transfection, the cells were treated with 5 nM ActD for 18 h followed by FACS analysis. *: *p* < 0.05. **E**. Analysis of human cancer database from cBioPortal reveals mutual exclusivity of RPL22/eL22 and TP53 gene mutations. p-Value: Derived from Fisher Exact Test. Log Odds Ratio: Quantifies how strongly the presence or absence of alterations in gene A are associated with the presence or absence of alterations in gene B in the selected tumors.

It has been shown that ribosomal stress, as a result of impaired ribosomal biogenesis caused by Actinomycin D, nutrient depletion and malfunction of nucleolar proteins involved in ribosome biogenesis, can lead to p53 activation [15]. To determine if RPL22/eL22 is required for ribosomal stress induction of p53, we employed three approaches to trigger ribosomal stress, e.g. knocking down RPL30/eL30[26] (Figure 3A), treatment with 5-FU[27] (Figure 3B) and treatment with low concentration of Actinomycin D (ActD) [28] (Figure 3C), in U2OS cells. As a result of RPL22/eL22 knockdown, ribosomal stress-induced activation of p53 and its target genes was markedly impeded (Figures 3A-3C). Consistently, ActD-induced G1/G0 cell cycle arrest was also significantly compromised when RPL22/eL22 was knocked down by its specific siRNA (Figure 3D). Notably, knockdown of RPL22/eL22 itself did not affect p53 level or the cell cycle (Figures 3A-3D). These results indicate that RPL22/eL22 is at least partially required for ribosomal stress activation of p53.

As mentioned above, RPL22/eL22 is highly mutated in several cancer types and a pool of cancer cell lines. Based on our observation that RPL22/eL22 plays a vital role in ribosomal stress induction of p53, we were curious about how RPL22/eL22 mutation is correlated with TP53 status in these cancers. Interestingly, analysis of the cBioPortal database revealed that RPL22/eL22 and TP53 mutations are mutually exclusive to each other in all of the 4 data sets with the highest RPL22/eL22 mutation rates (Figure 3E). This finding is consistent with a latest report (published right when we completed this manuscript), showing that RPL22/eL22 is the most recurrently deleted ribosomal protein gene in 30 cell lines with intact *TP53* [5]. These observations suggest that mutating RPL22/eL22 may be utilized by human cancers as a strategy to silence p53 response to ribosomal stress.

### RPL22/eL22 binds to MDM2 and suppresses MDM2-mediated p53 degradation

**Figure 4:**
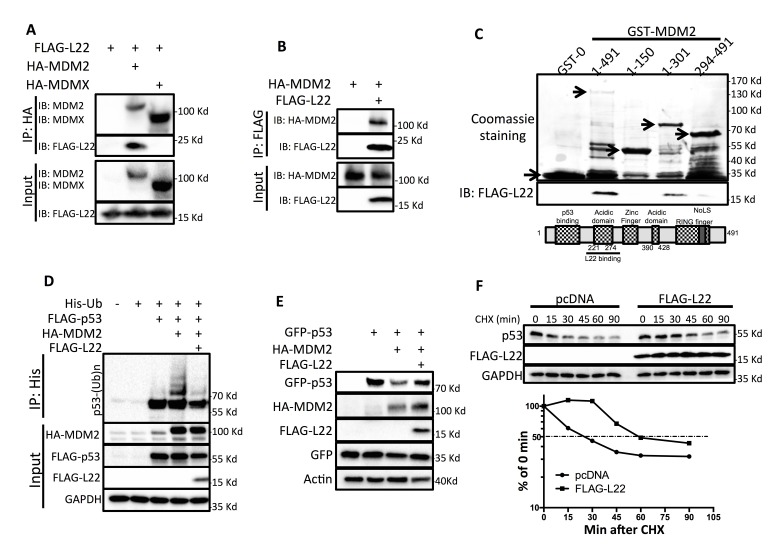
RPL22/eL22 binds to MDM2 and suppresses MDM2-mediated p53 degradation **A.** HEK293 cells were transfected with FLAG-L22 alone or FLAG-L22 plus HA-MDM2 or FLAG-L22 plus HA-MDMX and cell lysates were collected 48 h after transfection, followed by immunoprecipitation analysis using anti-HA antibody. **B**. HEK293 cells were transfected with HA-MDM2 alone or HA-MDM2 plus FLAG-L22 and cell lysates were collected 48 h after transfection, followed by immunoprecipitation analysis using anti-FLAG antibody. **C**. Purified GST alone, full-length GST-MDM2 (1-491), or GST-MDM2 deletion mutants including MDM2/1-150, MDM2/1-301, MDM2/294-491 immobilized on glutathione beads were used in GST pull-down assays with whole cell lysates containing ectopically expressed FLAG-L22. Bound L22 was detected by WB analysis with anti-FLAG antibody. **D**. H1299 cells were transfected with combinations of FLAG-L22, FLAG-p53, or HA-MDM2 constructs in the presence of the His-ubiquitin (His-Ub) plasmid as indicated. The cells were treated with MG132 for 6 h before harvesting. The in vivo ubiquitination assay was performed and ubiquitinated proteins were detected by WB analysis with indicated antibodies. **E.** H1299 cells were transfected with GFP-p53, or GFP-p53 plus HA-MDM2 in the absence or presence of FLAG-L22 and cell lysates were collected 48 h after transfection, followed by WB analysis with indicated antibodies. **F.** U2OS cells were transfected with pcDNA or FLAG-L22 for 48 h followed by addition of 50 mg/ml cycloheximide (CHX) and harvested at indicated time points for WB analysis with indicated antibodies. The intensity of each band was quantified, and normalized with GAPDH and plotted.

Our group and others have reported that inhibition of MDM2 by ribosomal proteins plays an important role in ribosomal stress induction of p53 [13-15]. To understand how RPL22/eL22 activates p53, and specifically, to determine if RPL22/eL22 activates p53 by inhibiting MDM2 activity like other p53-activating RPs, such as RPL11/uL5 or RPL5/uL18, we first performed co-immunoprecipitation (Co-IP) assays. As shown in Figure 4A, FLAG-L22 was only co-immunoprecipitated with HA-MDM2, but not HA-MDMX, when anti-HA antibody was used for Co-IP. Consistently, when anti-FLAG antibody was used for Co-IP, HA-MDM2 was co-immunoprecipitated with FLAG-L22 (Figure 4B), confirming the interaction between RPL22/eL22 and MDM2.

Next, we tried to map the RPL22/eL22-binding domain of MDM2 by performing a set of GST protein-protein binding assays with purified GST-MDM2 fusion proteins as shown in Figure 4C. As a result, we found that FLAG-RPL22/eL22 binds to the region that encompasses the central acidic domain (221-274), but not the N- or C-terminus, of MDM2 (Figure 4C). Since RPL5/uL18 binds to the same region of MDM2[29], we hypothesized that RPL22/eL22 may have similar function to what RPL5/uL18 does in suppression of MDM2 activity toward p53[13]. Indeed, MDM2-mediated p53 ubiquitination was drastically reduced by ectopic RPL22/eL22 (Figure 4D). Consistently, the degradation of GFP-p53 mediated by HA-MDM2 was partially rescued by ectopic RPL22/eL22 (Figure 4E). Consequently, the half-life of endogenous p53 in U2OS cells was prolonged from ∼30 min to ∼60 min by FLAG-L22 (Figure 4F). Together, these findings indicate that by binding to the central acidic domain of MDM2, RPL22/eL22 can suppress MDM2-mediated p53 ubiquitination and degradation, leading to p53 stabilization and consequent activation.

### RPL22/eL22 cooperates with RPL11/uL5 in activating p53

**Figure 5:**
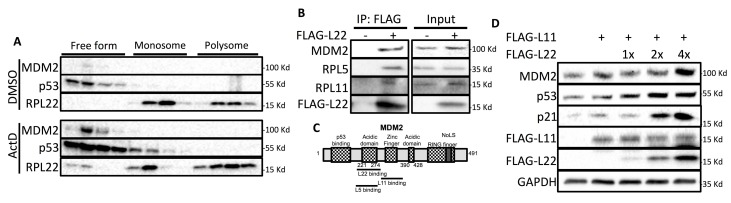
RPL22/eL22 forms complex with RPL5/uL18/RPL11/uL5/MDM2 and synergizes with RPL11/uL5 to activate p53 **A.** U2OS cells were treated with DMSO or 5nM ActD for 18 h and subjected to sucrose gradient fractionation analysis followed by WB analysis with antibodies as indicated. **B**. U2OS cells were transfected with pcDNA or FLAG-L22 and cell lysates were collected 48 h post-transfection, followed by immunoprecipitation analysis using anti-FLAG antibody. **C**. Structure depiction of MDM2 showing binding sites with RPL22/eL22, RPL11/uL5 or RPL5/uL18. **D**. U2OS cells were transfected with FLAG-RPL11/uL5 and increasing amount of FLAG-RPL22/eL22 and cell lysates were collected 48h post-transfection, followed by WB analysis with indicated antibodies.

In response to ribosomal stress, ribosomal proteins dissociated from ribosome become ribosome-free so that some of them, such as RPL5/uL18, or RPL11/uL5, can interact with MDM2 to achieve p53 activation [13, 14, 19, 30]. To test if RPL22/eL22 might also become non-assembled as ribosome-free form in response to ribosomal stress, we performed sucrose gradient fractionation analysis of U2OS cells after ActD treatment. Indeed, RPL22/eL22 was found in ribosome-free fractions (∼7% of all RPL22/eL22 detected) upon ActD treatment, accompanying the drastic increase of p53 and MDM2 protein levels as detected by WB analysis (Figure 5A). Interestingly, in addition to MDM2, FLAG-L22 also co-precipitated with endogenous RPL5/uL18 and RPL11/uL5 (Figure 5B), suggesting that they may form a complex in cells. Based on our observations (Figure 4C) and previous findings that the binding site of RPL22/eL22 on MDM2 overlaps with that of RPL5/uL18, but is adjacent to that of RPL11/uL5 (Figure 5C) [31], we wondered if RPL22/eL22 may have any synergistic effect with RPL5/uL18 or RPL11/uL5 on p53 activation. To address this question, we co-transfected U2OS cells with an equal amount of FLAG-L5 or FLAG-L11 and increasing amounts of FLAG-L22, followed by WB analysis. Interestingly, RPL22/eL22 cooperated with RPL11/uL5 (Figure 5D), but not RPL5/uL18 (Supplementary Figure 2), to induce p53 and MDM2 expression. These findings suggest that like other ribosomal proteins, RPL22/eL22 becomes non-assembled in response to ribosomal stress, and this ribosome-free RPL22/eL22, like RPL5/uL18 [32], cooperates with RPL11/uL5 to activate p53.

### The N- and C-termini of RPL22/eL22 play distinct roles in inhibiting MDM2

**Figure 6:**
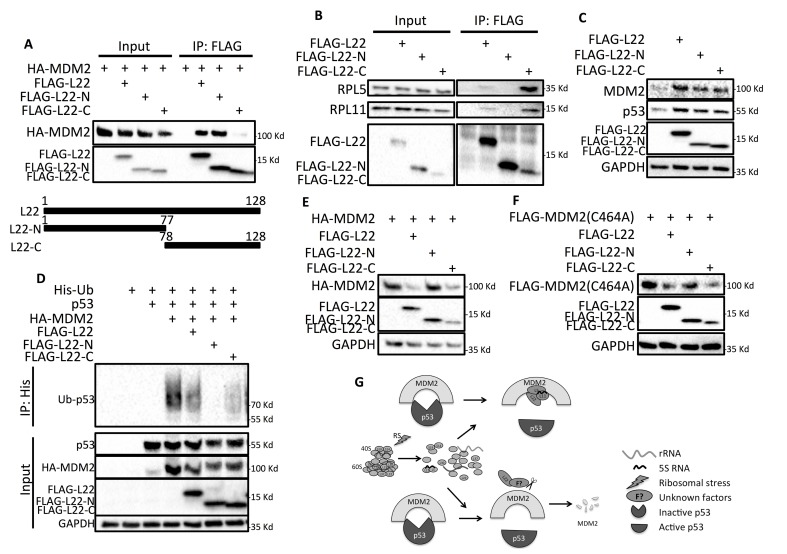
RPL22/eL22 N, C termini play distinct roles in inhibiting MDM2 **A.** H1299 cells were transfected with HA-MDM2 in the absence or presence of FLAG-L22, FLAG-L22-N or FLAG-L22-C, and 48 h later cell lysates were collected for IP analysis using anti-FLAG antibody. **B**. HCT116 p53-/- cells were transfected with FLAG-L22, FLAG-L22-N or FLAG-L22-C, and 48 h later cell lysates were collected for IP analysis using anti-FLAG antibody. **C**. HCT116 p53+/+ cells were transfected with FLAG-L22, FLAG-L22-N or FLAG-L22-C, and 48 h later cell lysates were collected for WB analysis with indicated antibodies. **D**. H1299 cells were transfected with combinations of FLAG-L22,FLAG-L22-N, FLAG-L22-C, FLAG-p53, or HA-MDM2 constructs in the presence of the His-ubiquitin (His-Ub) plasmid as indicated. The cells were treated with MG132 for 6 h before harvesting. The in vivo ubiquitination assay was performed and ubiquitinated proteins were detected by WB. **E**. & **F**. H1299 cells were transfected with FLAG-L22, FLAG-L22-N, FLAG-L22-C in the presence of HA-MDM2 (E) or FLAG-MDM2(C464A) (F), and cell lysates were collected for WB analysis with antibodies as indicated 48 h post-transfection. **G**. Proposed model depicting RPL22/eL22 activation of p53 through inhibition of MDM2.

To further decipher how exactly RPL22/eL22 inactivates MDM2 and consequently activates p53, we first generated two constructs covering the N- (amino acid 1-77) and the C-terminus (amino acid 78-128) of RPL22/eL22 as shown in Figure 6A to map its MDM2-binding domain. The structures of RPL22/eL22 N- and C- termini were shown in Supplementary Figure 3 based on RCSB Protein Data Bank (PDB) 5T2C [33]. The co-IP experiments clearly showed that the N-terminus of RPL22/eL22 binds to MDM2 (Figure 6A), while the C-terminus associates with endogenous RPL5/uL18 and RPL11/uL5 (Figure 6B). Surprisingly, although the binding partners were different, both of the N- and C-termini of RPL22/eL22 were able to activate p53 in HCT116^p53+/+^ cells (Figure 6C). Next, we tested if either or both of the N- and the C-termini of RPL22/eL22 can affect MDM2-mediated p53 ubiquitination by performing in vivo ubiquitination assays. As shown in Figure 6D, compared with the full length RPL22/eL22, its N-terminus demonstrated more potent inhibitory effect on MDM2-mediated p53 ubiquitination. Surprisingly, even though not binding to MDM2, the C-terminus of RPL22/eL22 also displayed drastic suppression of p53 ubiquitination, suggesting a different mechanism underlying MDM2 regulation by the C-terminus. Indeed, wild type FLAG-L22 and FLAG-L22-C, but not FLAG-L22-N, dramatically decreased the protein level of co-transfected HA-MDM2 (Figure 6E). However, down-regulation of MDM2 protein expression by FLAG-L22 and FLAG-L22-C was not affected by cysteine 464 mutation (C464A), a critical residue for MDM2 RING finger domain ubiquitin ligase activity [34] (Figure 6F). Collectively, these observations indicate that the N-terminus of RPL22/eL22 is responsible for MDM2 binding, which may eliminate MDM2 E3 ligase activity toward p53, whereas the C-terminus of RPL22/eL22 mediates interaction with RPL5/uL18/RPL11/uL5 and downregulates MDM2 protein expression independent of MDM2 E3 ligase activity.

## DISCUSSION

Although RPL22/eL22 was found to localize outside of the core-particle of 60S subunit and not essential for protein synthesis [25], the RNA-binding property may confer RPL22/eL22 important cellular functions. For instance, RPL22/eL22 can bind to Epstein-Barr virus (EBV) small RNA EBER1, and this binding is responsible for EBER1 growth-promoting capability in Akata Burkitt lymphoma cells [35, 36]. Also, in lymphoid precursors, RPL22/eL22 expression is transcriptionally activated by Miz-1 in response to V(D)J recombination, and elevated RPL22/eL22 subsequently binds to p53 mRNA to suppress its translation, preventing p53 from inducing cell death [7]. The regulation of p53 by RPL22/eL22 is also associated with T cell and B lymphocyte development, as RPL22/eL22 loss could impair the development of αβ T cell and B-lineage progenitors with p53 and multiple p53 target genes involved [6, 37]. However, these effects could be cell type specific or stage specific, because γδ-lineage T cells or splenic B cells were not impacted by RPL22/eL22-deficiency [6, 37].

Also, these findings do not explain why RPL22/eL22 is highly mutated in several human solid tumors [2, 3], as analysis of human cancer databases available from cBioPortal revealed that RPL22/eL22 is actually highly mutated in multiple cancer types and in a pool of cancer cell lines, and more than 85% mutations are deletion mutations (Figure 1). Interestingly, differing from other ribosomal protein genes whose deletion mutations are underrepresented in *TP53*-intact tumors due to negative-selection pressure, RPL22/eL22 deletion/mutation tends to occur in wild-type TP53 harboring tumors and cancer cell lines [5] (Figure 3E), further supporting its tumor suppressor role through activations of p53. Indeed, though ablation of RPL22/eL22 in mice did not cause lethal phenotypes [6], since this ribosomal protein is not essential for protein translation, knockdown of RPL22/eL22 clearly impaired p53 activation by ribosomal stress, indicating its requirement for ribosomal stress activation of p53 (Figures 3A-3D). Therefore, RPL22/eL22 mutation could be one of the strategies utilized by tumor cells to bypass p53 activation under stresses and also suggest that RPL22/eL22 might play a role in p53 regulation.

Based on our findings, we propose a model reflecting how RPL22/eL22 may contribute to p53 regulation through inhibition of MDM2 (Figure 6G). In response to ribosomal stress, RPL22/eL22 is not assembled into the large ribosome subunit, and the ribosome-free RPL22/eL22 then binds to the central acidic domain of MDM2 through its N-terminus in association with RPL5/uL18 and RPL11/uL5 through its C-terminus (Figures 4C, 6A & 6B). This ribosome-free multi-ribosomal proteins complex may constrain intrinsically disordered MDM2 [38] in a conformation that prevents MDM2 from promoting p53 ubiquitination and degradation, or blocking p53 transactivation activity, leading to activation of p53 (Figures 4D-4F and 6G). RPL22/eL22 binds to MDM2 at the central acidic domain (Figure 4C), which overlaps with that of RPL5/uL18 and is next to that of RPL11/uL5 [19, 29, 30] (Figure 5C). The difference in binding positions may explain the result that cooperative activation of p53 only occurs between RPL22/eL22 and RPL11/uL5, but not between RPL22/eL22 and RPL5/uL18 (Figure 5D & Supplementary Figure 2). This also suggests that binding between RPL22/eL22 and RPL11/uL5 is likely direct, while binding between RPL22/eL22 and RPL5/uL18 might be indirect. More biochemical and biophysical studies are necessary to further dissect the functional association between these ribosomal proteins in regulation of MDM2.

Intriguingly, despite the fact that only the N-terminus of RPL22/eL22 binds to MDM2, both the N- and C-termini could activate p53 via suppression of MDM2-mediated p53 ubiquitination (Figures 6C & 6D). Further investigation indicated that without altering mRNA expression of MDM2 (data not shown), the RPL22/eL22 C-terminal domain could decrease MDM2 protein expression independent of MDM2 E3 ligase activity, as evidenced by similar reduction of wild type MDM2 and MDM2 C464A mutant, an E3 ligase inactivation mutation [34] (Figure 6E & 6F). Thus, it is less likely that RPL22/eL22-mediated MDM2 protein downregulation is through MDM2 auto-ubiquitination. We therefore also propose that RPL22/eL22 could recruit a yet unknown factor(s) to facilitate MDM2 protein downregulation, resulting in p53 accumulation and activation (Figure 6G). Identification of the tentative molecule(s) recruited by RPL22/eL22 to negatively regulate MDM2 protein expression is of great interest for our future studies. Furthermore, the ability of RPL22/eL22 to decrease MDM2 expression at protein level may also serve as a molecular base for developing drugs targeting MDM2 for protein downregulation to abrogate its multiple oncogenic functions[39-41]. Our findings that the N- and C-termini of RPL22/eL22 play distinct roles in MDM2 inhibition may also explain why most of the RPL22/eL22 mutations are deletion mutations, as otherwise the inhibitory effect on MDM2 by different domains of RPL22/eL22 may not be eliminated completely in cancer cells.

Hence, our study as presented here not only delineates the detailed mechanism by which RPL22/eL22 suppresses cancer cell survival by blocking the MDM2-p53 feedback loop and consequently activating p53 probably as part of the ribosome-free ribosomal protein sub-complex, but also provides a new molecular insight into understanding clinical relevance of high mutation rate of RPL22/eL22 in some human cancers.

## MATERIALS AND METHODS

### Cell lines

Human HCT116 p53^+/+^ and HCT116 p53^-/-^ cells were generous gifts from Dr. Bert Vogelstein at the John Hopkins Medical Institutes. U2OS, H460 and H1299 cells were purchased from American Type Culture Collection (ATCC). All cells were cultured in Dulbecco’s modified Eagle’s medium (DMEM) supplemented with 10% fetal bovine serum, 50 U/ml penicillin and 0.1 mg/ml streptomycin at 37 °C in 5% CO_2_.

### Plasmids and antibodies

FLAG-L22 was generated by inserting RPL22/eL22 cDNA into 2FLAG-pcDNA3 at BamHI and EcoRI sites. The primers used for PCR amplifying reverse transcribed mRNA were: Forward-CGGGATCCatggctcctgtgaaaaagcttg; Reverse-GGAATTCttaatcctcgtcttcctcctct. FLAG-L22 was used as template to generate RPL22/eL22 N and C terminal fragments, and the primers were as follows: FLAG-L22-N Forward- CGGGATCCatggctcctgtgaaaaagcttg, FLAG-L22-N Reverse- GGAATTCttaaggcacctcggatgtcac; FLAG-L22-C Forward- CGGGATCCatgtccgaggtgcctttctcc, FLAG-L22-C Reverse- GGAATTCttaatcctcgtcttcctcctct. HA-MDM2, HA-MDMX, FLAG-p53, GFP-p53, His-Ub, FLAG-L5, FLAG-L11, and FLAG-MDM2 (C464A) mammalian expression plasmids and the GST-MDM2 and its fragments were described previously [13, 14, 26]. The anti-p21, anti-p53, anti-RPL30/eL30, and anti-RPL22/eL22 antibodies were purchased from Santa Cruz. The anti-GAPDH antibody was from Millipore. The anti-RPL5/uL18 [13], anti-RPL11/uL5 [42], and anti-MDM2 [13, 14] (2A10 and 4B11) antibodies were described previously.

### Transient transfection, Western blotting and immunoprecipitation

Cells were transfected with plasmids as indicated in the figures using TurboFect reagent as per the manufacturer’s instruction (Themo Scientific). Cells were harvested at 30-48h post transfection and lysed in lysis buffer consisting of 50 mM Tris/HCl (pH7.5), 0.5% Nonidet P-40 (NP-40), 1 mM EDTA, 150 mM NaCl, 1 mM phenylmethylsulfonyl fluoride, 0.25 mg/ml pepstatin A and 1 mM leupeptin. Equal amounts of clear cell lysates (20-50 μg protein) were used for WB analysis as described previously. IP was conducted using antibodies indicated in the figures and described previously. Beads were washed twice with lysis buffer and once with RIPA buffer (50 mM Tris/HCl pH8.0, 5 mM EDTA, 1% NP-40, 0.5% Deoxycholate, 0.1% SDS, 150 mM NaCl). Bound proteins were detected by WB with indicated antibodies.

### *In vivo* ubiquitination assay

The *in vivo* ubiquitination assay was performed as previously described with minor modification [14, 43]. Briefly, H1299 cells were transfected with plasmids as indicated. 42 h after transfection, cells were treated with 20 μM MG132 for 6 h, and then collected in two aliquots, one lysed in lysis buffer for WB analysis for input detection, and the other lysed in buffer I (6 M guanidinium-HCl, 0.1 M Na_2_HPO_4_/NaH_2_PO_4_, 10 mM Tris-HCl (pH 8.0), 10 mM β–mercaptoethanol) and incubated with Ni-NTA beads (QIAGEN) at room temperature for 4 h. Beads were washed once with buffer I, buffer II (8 M urea, 0.1 M Na_2_HPO_4_/NaH2PO_4_, 10 mM Tris-HCl (pH 8.0), 10 mM β–mercaptoethanol), and buffer III (8 M urea, 0.1 M Na_2_HPO4/NaH_2_PO4, 10 mM Tris-HCl (pH 6.3), β–mercaptoethanol). Proteins were eluted from beads in buffer IV (200 mM imidazole, 0.15 M Tris-HCl (pH6.7), 30% glycerol, 0.72 M β–mercaptoethanol, and 5% SDS) and subjected to WB analysis.

### RNA interference

The siRNA against RPL22/eL22, RPL5/uL18, RPL11/uL5, and RPL30/eL30 were purchased from Ambion. 20-40 nM siRNA were introduced into cells using TurboFect according to manufacturer’s protocol. Forty eight hours later, cells were treated with drug for 18 h, followed by WB or flow cytometry analysis, or transfected with indicated plasmids, followed by WB 30-48 h after transfection.

### Flow cytometry analysis

Cells were fixed and stained in 500 μl propidium iodide (PI, Sigma-Aldrich) stain buffer (50 mg/ml PI, 200 mg/ml RNase, 0.1% Triton X-100 in phosphate-buffered saline) at 37 °C for 30 min. The cells were then analyzed fro DNA content using a FACScan flow cytometer (BD Biosciences, San Jose, CA, USA). Data were analyzed using the CellQuest (BD Biosciences) and Modifit (Verity, Topsham, ME, USA) software.

### Sucrose gradient fractionation and ribosome profiling

The assay was performed following the protocol previously reported [44]. Briefly, cells were harvested at 70-80% confluence after halting translation by 100 μg/ml cycloheximide incubation for 10 min. Cells were lysed in lysis buffer (10 mM Tris-HCl pH7.4, 5 mM MgCl_2_, 100 mM KCl, 1% Triton X-100) and gently sheared with a 26-gauge needle for 4 times. Lysates were subjected to 10-50% sucrose gradient centrifugation and the fractions were collected through BR-188 Density Gradient Fractionation System (Brandel, Gaithersburg, MD, USA).

### Statistical analysis

The Student’s two-tailed t test was used to compare the mean differences between treatment and control groups, unless otherwise indicated. Data are presented as Mean ± SD (standard deviation). *p* < 0.05 was determined as statistically significant.

## SUPPLEMENTARY MATERIALS FIGURES


