# Supplementary material for: Cancer-mutated ribosome protein L22 (RPL22/eL22) suppresses cancer cell survival by blocking p53-MDM2 circuit
Source: Oncotarget. 2017 Oct 6;8(53):90651–61. doi: 10.18632/oncotarget.21544 (PMC5710875; doi:10.18632/oncotarget.21544)
Supplement: Supplementary file 1 [file oncotarget-08-90651-s001.pdf]

Cancer-mutated ribosome protein L22 (RPL22/eL22) suppresses cancer cell survival by blocking p53-MDM2 circuit

SUPPLEMENTARY MATERIALS

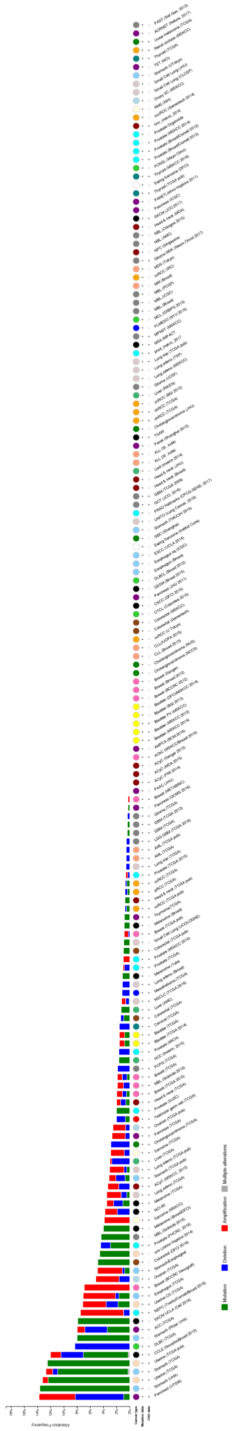

Supplementary Figure 1: Mutation and copy number analysis of RPL22/eL22 are retrieved from cBioportal with all available data sets included.

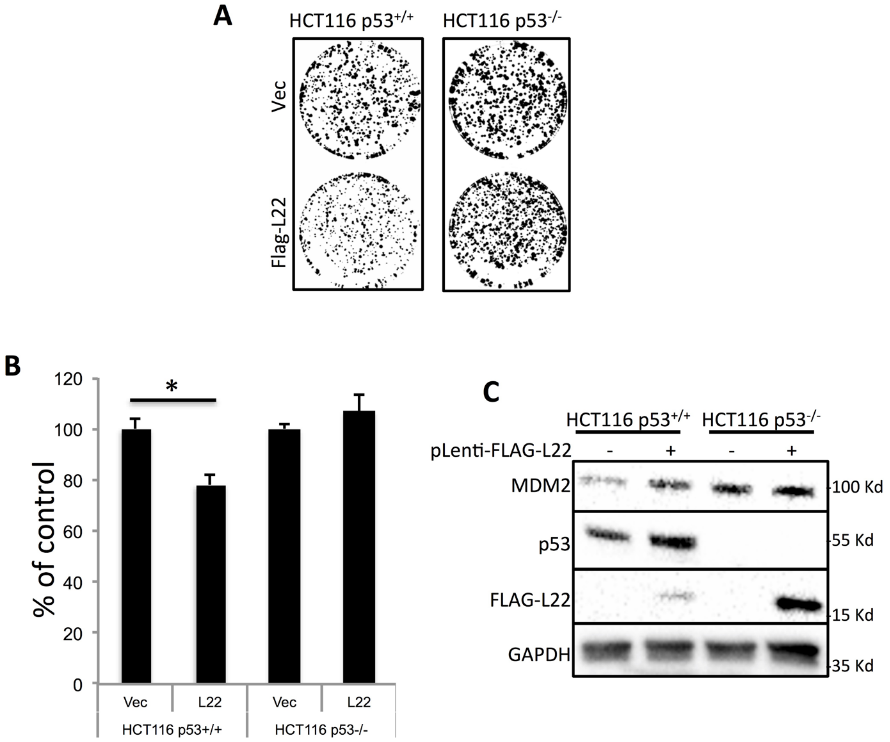

Supplementary Figure 2: HCT116 p53+/+ and HCT116 p53-/- cells were infected with pLenti-vector (Vec) or pLenti-FLAG-L22/eL22 (FLAG-L22) and subjected to colony formation assay. Representative plates are shown in **A**. Quantification of triplicate experiments is shown in **B**. *, $p<0.05$ as compared to a vector control. Activation of the p53 pathway was confirmed by WB analysis with antibodies as indicated in **C**.

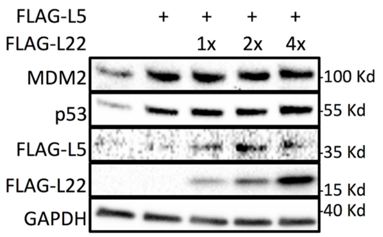

Supplementary Figure 3: U2OS cells were transfected with FLAG-RPL5/uL18 and increasing amounts of FLAG-RPL22/eL22. Cell lysates were collected 48h posttransfection, followed by WB analysis with antibodies as indicated.

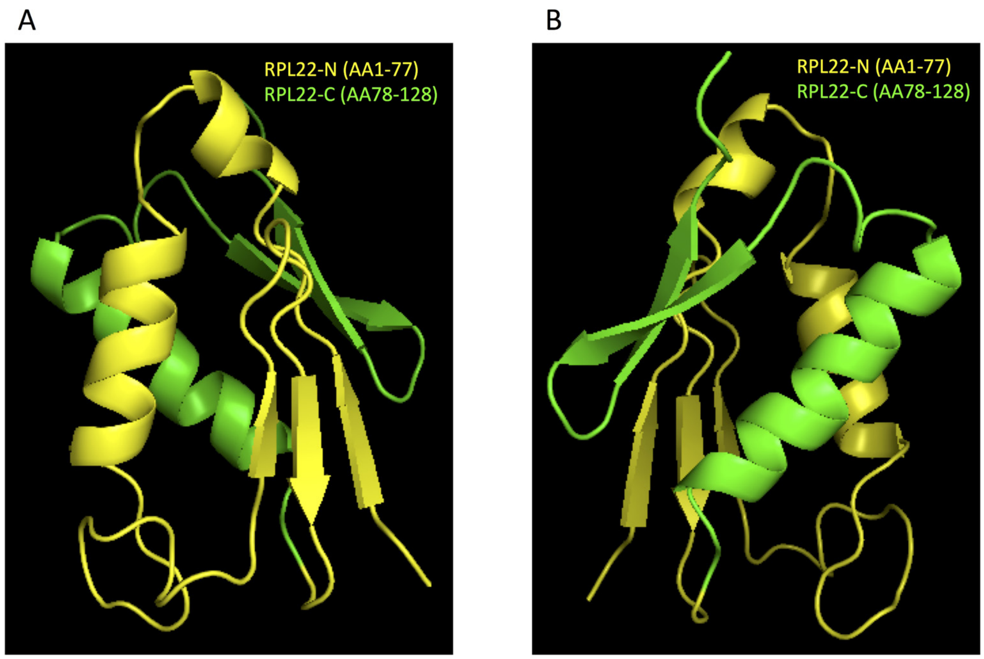

Supplementary Figure 4: Cartoon style RPL22/eL22 structure derived from PDB 5T2C using PyMol software (Ref 1). **A**. RPL22/eL22-N terminus (yellow) is pictured in the forefront. **B**. RPL22/eL22-C terminus (green) is pictured in the forefront.

REFERENCES

1. Zhang X, Lai M, Chang W, Yu I, Ding K, Mrazek J, Ng HL, Yang OO, Maslov DA, Zhou ZH. Structures and stabilization of kinetoplastidspecific split rRNAs revealed by comparing leishmanial and human ribosomes. Nat Commun 2016;7: 13223.
